# Lessons learned from the approach to the COVID-19 pandemic in urban primary health care centres in Barcelona, Spain

**DOI:** 10.1080/13814788.2020.1796962

**Published:** 2020-07-27

**Authors:** Miguel-Angel Muñoz, Mercè López-Grau

**Affiliations:** a Gerencia Territorial de Barcelona, Institut Català de la Salut, Barcelona, Spain; b Fundació Institut Universitari per a la recerca a l’Atenció Primària de Salut Jordi Gol i Gurina (IDIAPJGol), Barcelona, Spain; c Departament de Pediatria, Obstetricia i Ginecologia, i Medicina Preventiva, Universitat Autònoma de Barcelona, Bellaterra (Cerdanyola del Vallès), Spain; d Primary Healthcare Centre Passeig de Sant Joan, Institut Català de la Salut, Barcelona, Spain

The recently published article by de Sutter et al. [1] reflects the transformation that the coronavirus epidemic has caused in our world. With the need to adapt to the novel reality, new challenges and tasks for primary healthcare (PC) professionals have arisen. We will present here how PC has dealt with the issues emerging as the aftermath of the disease in Barcelona, Spain.

## How the pandemic affected primary care and its response

The organisation of PC varies across Europe. In the Spanish urban setting, it is managed in large primary healthcare centres (PHC), which act as gatekeepers providing services to an average of 25,000 inhabitants. Citizens are assigned to a team consisting of a General Practitioner (GP) and PC nurse who manages a list of around 1,300 patients.

The coronavirus initially appeared in Barcelona on 25th February 2020. During the first weeks of the pandemic, several patients visited the PHC with symptoms compatible with COVID-19; nevertheless, they did not fit the epidemiologic criteria used at that moment to be considered as possible cases. As a consequence, healthcare professionals did not protect themselves appropriately. This might be one of the reasons that by 21st May 2020, a total of 40,921 healthcare professionals had been infected and 53 had died in Spain [2].

With the arrival of COVID-19, everything in PC organisation changed. Patients were afraid to attend the PHC, and GPs and nurses were instructed to avoid as much physical contact as possible with them. Triage by telephone or telemedicine/online was provided to reduce the number of contacts of the symptomatic population following the recommendations of the European Centre for Disease Prevention and Control [3].

Clinical examinations were restricted to those cases considered unavoidable, and invisible and visible barriers were constructed between the population and healthcare professionals. Separated areas were created to attend patients with respiratory symptoms and healthcare professionals were instructed to adopt the highest measures of safety and protection.

Nevertheless, masks and adequate personal protective equipment were not always available. Subsequently, in many cases, GPs and nurses had to wear improvised face shields made by themselves. Scientific evidence was quick in emerging, and the changing rules and recommendations confused professionals about the best way to approach the disease.

However, the challenge was faced and patients were attended.

## Why was primary care crucial during the worst period of the outbreak?

The organisation of the PHC needed to adapt to the new scenario. The number of patients attending the centres decreased dramatically whilst the number of virtual consultations *via* email and telephone increased. Among the 2,127,610 consultations attended in the PHC between 1st March and 7th June in Barcelona, 1,453,666 (68.3%) were solved in this way. Also, healthcare professionals visited their patients at home 72,825 times. The PHC also opened at weekends to improve accessibility.

PC has been shown to be essential in early diagnosis. Patients who consulted the healthcare professionals due to fever, coughing or breathlessness were assessed by clinical exploration and oxygen saturation. GPs advised patients to either return home (with or without COVID-19 diagnosis) or referred them to the hospital if they presented severe symptoms and signs.

In the case that symptoms persisted or worsened, patients were instructed to consult their GPs and then directly referred to the radiology service for a chest X-ray.

Patients identified as COVID-19 had a telephone follow-up with PC professionals every 24, 48 or 72 h, depending on their clinical status. Patients and families were taught to manage symptoms, implement isolation and hygienic measures, and encouraged to call the PHC in the case of worsening. A total of 12,497 and 28,799 patients were registered in PC records as a confirmed or possible coronavirus infection, respectively, and 122,479 follow-up consultations were made with these patients between 1st March and 7th June.

Elderly residents in 200 nursing homes located in Barcelona were most affected by COVID-19, leading to high morbidity and mortality. PHC professionals took care of this population by detecting infections, recommending isolation, or referring patients to hospital if necessary. In addition, they assisted those experiencing the final stages of the disease by providing end of life care. One in three older adults living in these facilities was affected by the disease, and 31,877 consultations were attended by GPs, primary care nurses, and social workers.

Patients with social problems regarding proper home isolation, and those cohabiting with vulnerable individuals (the immunosuppressed, individuals with multimorbidity or other risk factors), were accommodated in six hotels adapted by the Department of Health in Barcelona.

One of the most important tasks of the PC professionals was the emotional support they offered to the families and patients. COVID-19 originated an unprecedented situation, which resulted in patients’ isolation at home or hospital and exacerbated feelings of loneliness and mental distress.
